# ‘Let food be thy medicine…’: lessons from low-protein diets from around the world

**DOI:** 10.1186/s12882-017-0515-8

**Published:** 2017-03-27

**Authors:** Giorgina B. Piccoli, Adamasco Cupisti

**Affiliations:** 1 Department of Clinical and Biological Sciences, University of Torino, Torino, Italy; 2 Nephrologie, CH Le Mans, Le Mans, 72000 France; 3 Department of Clinical and Experimental Medicine, University of Pisa, Pisa, Italy

**Keywords:** Chronic kidney disease, Low-protein diets, Dialysis start, Nutritional status, Dialysis

## Abstract

In this editorial we present the special issue dedicated to low-protein diets (LPDs) in chronic kidney disease, from a global perspective.

The experiences gathered from several countries across all continents have created an issue which we hope you will find insightful, and lead to further discussion on this interesting topic.

We discover that LPDs are feasible in both developed and low income countries, in patients where literacy is an issue, and are also safe, including during pregnancy and in old age.

Patients prescribed a low protein diet are more inclined to follow and adhere to this change in lifestyle, provided the diet has been adapted to meet their own individual needs. With an increasing list of different menu options and better medical advice being offered we no longer need to identify low protein diets with a specific menu, ingredient or supplement, or with a specific level of protein restriction. Evidence shows how the best diet is often the one chosen by the patients, which doesn’t drastically affect their day-to-day life, and delays the start of dialysis for as long as is safe under careful clinical control. The colourful menus gathered from all over the world remind us that a low protein diet does not necessarily mean that the pleasure of preparing a delicious meal is lost. The final comment is therefore dedicated to our patients: low protein diets can be beautiful.

## An overall view: low-protein diets, and changes in eating habits

There have been enormous advances in medicine in the new millennium: dozens of new drugs have come onto the market; new diagnostic approaches have been developed; new genes have been associated with their functions, and the secrets of the human genome are being progressively unraveled. Yet, despite the progress that has been made in so many fields, we still know far too little about nutrition, the basis of human life.

**Fig. 1:**
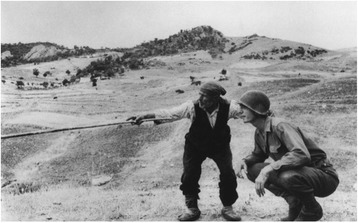
Robert Capa. Near Troina. August 4-5, 1943. Sicilian farmer showing an American officer which way German soldiers have gone (source: http://www.slideshare.net/); the permission for reproducing the photo was obtained from right holder at Magnum Photos (http://www.magnumphotos.com/).

Photographer Robert Capa, took an image during the Second World War which depicts an extremely tall, young American soldier who is squatting at the side of a very short, ageless Italian shepherd to try and see from his perspective. This may be considered an example of how different genetic and nutritional backgrounds combine to produce divergent phenotypes (Fig. 1). Indeed, it was only after the Second World War that a new era of investigating nutrition and diets, as determinants of health and disease became popular.

The Seven Countries Study (SCS) was the first major study to investigate diet and lifestyle along with other risk factors such as cardiovascular disease, by obtaining data from Mediterranean and Northern European countries, Japan and the USA. The study’s findings catalyzed previous intuitions and brought about a new theory on the role of the diet in determining the health of a population and, in particular, the incidence of cardiovascular diseases and neoplasia [1].

To cite this study, in the late 1940s, seemingly healthy middle-aged men were “dropping dead in the streets of America”, while poor Italian workers survived in the streets of Naples [1]. Modern diet epidemiology started with SCS, which brought about the concept of health populations, the link between cholesterol levels and cardiovascular diseases, the relationship between physical activity and blood levels of several nutrients. Curiously, while evidence about the advantages of plant-based, Mediterranean diets were accumulating, the world population moved to higher protein intake, which was only recently reduced, also on account of the emerging data on the negative effects of diets particularly rich in red meat and processed food. Only relatively recently have we seen the rediscovery of the Mediterranean diet, poor in red meat and rich in oil rather than animal fats, and of several other plant-based diets and traditional foods, offsetting the trend towards quickly prepared, packaged, unhealthy food [2–8].

The SCS taught us the importance of adapting to local habits, and suggested that the best results can best be obtained by encouraging the consumption of traditional local foods: dark chocolate in the Netherlands, olive oil in Italy and Greece, and raw fish in Japan can “protect health” [1].

The kidney is a faithful mirror of the vascular tree and what is good for the heart is good for the kidneys. Therefore, a Mediterranean or plant-based diet might be a good starting point for a renal diet and studying patients’ backgrounds and their cultures’ traditional “healthy foods” may help to find feasible diet options.

This special issue was born in such a context.

## Low-protein diets from a global perspective

The Seven Countries Study takes us back to the aftermath of the second-world-war when, almost paradoxically, cultural exchanges had increased as a result of the war. The desire to share information about the advances in medical knowledge, made during and after the war was widespread. Meanwhile, the interest in diet and nutrition was modulated by the complex social and cultural changes, most recently merging into the on-going world crisis, globalization, and the aging of the overall population [9–12].

Nephrology progressed at an incredible speed; dialysis became possible, then widely available in western countries, and increasingly required in aging populations. Fifty years since the beginning of maintenance dialysis treatments, the issue of patient selection for dialysis is being posed again on a global scale. Since dialysis is not available or affordable for almost two-thirds of the world’s population and, even in rich countries, the limited resources open interrogatives on the indication for treatment in very old or severely comorbid patients [13].

In low-income countries, the use of well-balanced, low protein diets (LPD) may serve to prolong survival for a few years in patients who have no access to dialysis: it is a not quite sufficient, albeit not negligible, goal when dialysis is missing.

This problem is discussed in this issue.

The programs Remuzzi designed to improve the survival of patients with AKI or CKD in developing countries demonstrated that simple, low-cost management approaches could be used to slow the progression of CKD in thousands of individuals [14].

Other papers deal with this important question. The African experience shows that a well-balanced diet, with a restricted protein content, can be proposed in many contexts, including low-income, low-literacy settings [15, 16]. Given the “silent epidemic” of illiteracy in developed countries, the strategies developed in Africa can be used in Europe, as an Italian case report explains [17].

The policy of “dialysis early, dialysis for all”, has now been replaced by the “intention to delay” and by the consideration that it does not always improve survival, especially in very elderly or severely comorbid patients [18–20]. Consequently, interest has shifted to the effect of low-protein diets to delaying the progression of CKD, to their potential role as metabolic stabilizers, allowing a longer period of acceptable clinical balance without dialysis, and finally to the feasibility and implementation strategies [21, 22].

One consequence of the recent interest in incremental dialysis has been to suggest a role of LPDs in allowing smoother dialysis start and in preserving residual renal function, one of the strongest predictors of outcome [23–25].

## What else can we learn from this issue?

Following Mitch and Remuzzi’s commentary, which reconsiders their pivotal paper “Diets For Patients With Chronic Kidney Disease, Still Worth Prescribing”, many new and interesting aspects of low protein diets have emerged in the past decade [14, 20]. Their paper deals with the recent evidence on some complex aspects that often make the findings of studies on low-protein diets difficult to evaluate, among them the extent to which they interact with other aspects of the global care of CKD patients, such as metabolic acidosis or hyperphosphatemia. While the authors focus on high-quality evidence from large trials, they also mention how observational studies also represent important tools for implementation in clinical practice.

This is the underlying belief of the articles you are about to read.

The various experiences here presented convey several different messages, and may also dispel common beliefs associated with low protein diets.

In summary:

- Low protein diets are feasible.
- Low protein diets are safe and may be followed with good adherence.
- Low protein diets are adaptable to different settings and may be designed with different “menus”.
- Low protein diets do not have to be boring.
- Low protein diets are not restricted to any geographic location or alimentary habit.
- Low-protein diets can be delicious.

## Low-protein diets are feasible

Low-protein diets are feasible everywhere: in countries like Italy, in which several alternative options have been developed over time, as well as in Countries such as Japan, India, Brazil, Russia Sweden and Romania, in which fewer options are routinely followed [21–27].

Evidence exists that low-protein diets are safe, provided that they are correctly prescribed, and patients receive regular check-ups to ensure they are following the indications they have been given. This is a leitmotif in all the papers, which underline the importance of careful clinical control of on-diet patients, whenever possible by both a nephrologist and a renal dietician or, even better, by a larger work team [28–41].

Such diets should be adapted to suit local environment, culture, and religious practices, in addition to the single patient’s needs. Adaptation to local habits, and, within them, to the individual patient’s needs, appears to be the key to success. In countries such as Australia or the US, in which the normal protein intake is well above 1.2 g of protein per kg of body weight per day, a feasible dietary protein reduction would probably be a protein intake of 0.8 g/kg/day. A similar intake may be needed in poorer countries, for balancing malnutrition [16, 32, 33].

The availability of amino acid and ketoacid supplements helps to simplify diet strategies, and their use makes it possible to safely reduce protein intake to 0.3 g/Kg/day, at least in some patients. The European and Asian experiences are remarkably positive, both with low- and very low-protein regimens, even for prolonged periods of time [26, 35].

The evidence of the favorable role of moderately restricted vegan supplemented diets on fetal growth is an important element demonstrating the safety of this approach, as long as there is careful multidisciplinary monitoring [36].

The Italian experience with protein-free foods is of particular interest. These products, available free of charge only in this country, represent an easy way to increase energy intake without raising the dietary load of nitrogen, phosphate or potassium, which suggests that this interesting option should be made more widely available [21–24].

The common error of identifying low-protein diets with specific supplements, menus or a given degree of restriction may be clearly avoided by acknowledging the wealth of recipes, suggestions, and approaches presented, once more underlining the need to adapt diets to patients’ needs and wishes.

Thus, this issue stresses that “the best diet” is one that the individual patient agrees to follow and allows him/her to maintain an active social life, while correcting metabolic abnormalities and delaying the start of dialysis, as long and safely as possible. The colorful menus gathered from around the world describe not only feasible, but also wonderful diets with delicious dishes, and show that reducing protein intake need not entail reducing the pleasure of eating well-prepared, tasty food.

## Conclusions

In conclusion, we consider that this issue shows that we should stop seeing low-protein diets as being lists of “restricted or forbidden foods” and that we should conversely start offering our patients choices of “unrestricted and allowed foods”.

The final message is therefore for our patients: low-protein diets are effective and safe, but some effort must be made to make them tasty and easy to follow.

What you eat can be delicious, even on a low-protein diet.
